# Erector Spinae Plane Block for Different Laparoscopic Abdominal Surgeries: Case Series

**DOI:** 10.1155/2018/3947281

**Published:** 2018-02-18

**Authors:** Serkan Tulgar, Onur Selvi, Mahmut Sertan Kapakli

**Affiliations:** ^1^Department of Anesthesiology and Reanimation, Maltepe University Faculty of Medicine, Istanbul, Turkey; ^2^Department of General Surgery, Maltepe University Faculty of Medicine, Istanbul, Turkey

## Abstract

The ultrasound guided erector spinae plane (ESP) block is a recent block described for various surgeries for postoperative analgesia. ESP block has effect on both visceral and somatic pain; therefore, its use in laparoscopic cholecystectomy and other abdominal surgeries can be advantageous. We describe successful ESP block application in three different cases for postoperative pain. Two patient were operated on using endoscopic retrograde cholangiopancreatography and laparoscopic cholecystectomy and one patient was operated on using laparoscopic cholecystectomy together with the inguinal hernia operation.

## 1. Introduction

Ultrasound guided erector spinae (ESP) block is a regional anesthesia technique, recently described by Forero et al. [1] for use in thoracic neuropathic pain. ESP block is reported to lead to analgesic effect on somatic and visceral pain by effecting the ventral rami and rami communicantes that include sympathetic nerve fibers, as LA spreads through the paravertebral space [1, 2]. When performed bilaterally it has been reported to be as effective as thoracic epidural analgesia [2].

ESP block leads to effective postoperative analgesia when performed at T 4-5 level for breast and thoracic surgery, and T 7 level for abdominal surgeries [2–4]. The number of surgeries involving multiple procedures and/or incisions is increasing [5], with such surgeries requiring complex analgesia protocols for pain management.

As LA widely spreads cranially and caudally when ESP is performed, we hypothesized that ESP can effectively be used as an analgesic method for abdominal surgeries especially those involving more than one procedure and/or incision in a single session. The effectiveness of ESP block as an analgesic method in laparoscopic cholecystectomy (LC), endoscopic retrograde cholangiopancreatography (ERCP), and laparoscopic inguinal hernia repair has not been reported previously.

Herein we report three patients undergoing multiple abdominal procedures in a single surgical session in which ESP was successfully performed for postoperative analgesia.

## 2. Case Reports

Written informed consent was obtained from patients for this report. Ethics board approval for case reports was not required by our institute.

### 2.1. Patient 1

A 48-year-old female patient (weight: 66 kg, height: 166 cm) with multiple millimetric gallstones was due to undergo intraoperative ERCP followed by LC. She had a history of caesarean section and appendectomy and had elevated transaminases plus hyperbilirubinemia. She was accepted as being American Anesthesiology Association (ASA) Class 1. ESP block was planned as part of her multimodal analgesia protocol.

After premedication (midazolam 1 mg), anesthesia induction was performed using lidocaine 1 mg/kg, fentanyl 100 mcg, propofol 3 mg/kg, and rocuronium bromide 0.6 mg/kg. Following intubation, anesthesia was maintained with 0.6 MAC sevoflurane in air-oxygen mixture and remifentanil infusion of 0.08–0.1*μ*g/kg/min. After hemodynamic stability, the patient was placed in the prone position. Bilateral ESP block was performed. After completing ERCP, the patient was positioned supine and LC was performed. Total surgical time was 74 minutes and time under anesthesia was 93 minutes. Perioperative intravenous paracetamol (1 gr) and tenoxicam (20 mg) were given.

She was transferred to the postoperative recovery room after extubation. The patients numeric rating scale (NRS) was 1/10 at rest and when coughing. After follow-up of 1 hour the patient was transferred to the general ward. Postoperative analgesia was ordered as 1 gr intravenous paracetamol every 8 hours. Rescue analgesia was planned as being intramuscular diclofenac sodium 75 mg. The patient's NRS was <3/10 during the first 16 hours of follow-up. Planned analgesia was not applied during this time. At 17th hour, NRS scores were 5/10 when coughing and 4/10 at rest. Rescue analgesia was performed. The patient was externalised home at the 24th hour with prescription pain medication.

### 2.2. Patient 2

A 54-year-old male patient with multiple millimetric gallstones was due to undergo intraoperative ERCP followed by LC. The patient had a history of smoking 35 pack/years, weighed 84 kg, and was 176 cm tall and ASA class 2. The patient underwent endoscopic sphincterotomy. If ERCP failed, it would go through open surgery. After a successful ERCP, the process was continued with LC. He had high liver function tests, hyperbilirubinemia, and jaundice. Anesthesia, analgesia, and surgical plans were the same as Patient 1. Surgical time and anesthesia time were 92 and 108 minutes, respectively.

In the recovery room, the patients NRS on coughing and at rest was 1/10. Patients NRS was <3/10 during first 12 hours of follow-up. No analgesic medication was applied during this time. On 13th hour, NRS was found to be 5/10 on coughing and 3/10 at rest. Rescue analgesic was performed. The patient was externalised home at the 24th hour with prescription pain medication.

### 2.3. Patient 3

A 52-year-old male patient was due to undergo LC and right laparoscopic inguinal hernia repair (Transabdominal preperitoneal repair technique). The patient had a history of smoking 30 pack/years and had hypertension. He weighed 96 kg and was 169 cm tall. Anesthesia, perioperative and postoperative analgesia, and ESP block were the same as Patient 1. LC followed by laparoscopic inguinal hernia repair was performed in the supine position. Due to two surgical procedures and the length of surgical time (152 minute), ESP block was added to the multimodal analgesia. The patient was placed in the lateral position and ESP was performed. Total time under anesthesia was 163 minutes. The patients received perioperative 1 gr paracetamol, 20 mg tenoxicam, and 100 mg tramadol intravenously.

The patient's NRS in the recovery room was 5/10 at rest and when coughing. Intravenous fentanyl 25 mg was given. At 20th minute, NRS was found to have decreased to 1/10. He was transferred to the general ward after 1 hour. No additional analgesic was required for the first 15 hours (NRS < 3/10). At 16th hour NRS was found to be 5/10 when coughing and 4/10 at rest. Rescue analgesic was given. Follow-up was discontinued after 24 hours.

No nausea or vomiting was observed in all patients.

### 2.4. Performing the ESP Blocks

Local anesthetics mixtures used in ESP blocks were prepared as 20 ml including ten ml bupivacaine 0.5%, five ml lidocaine 2%, and five ml serum physiologic. 

*Patient 1-2*. After anesthesia induction, the patient was placed in prone position for ERCP. Under aseptic conditions, a high frequency linear transducer was placed on the spinous process at T8 level on the parasagittal plane and then slid 2.5–3 cm laterally to visualise the transverse process and erector spinae muscle. Using the in plane technique, the needle was advanced between the transverse process and erector spinae muscle. The correct location was confirmed using 1 ml of LA to view hydrodissection. 19 ml of LA was injected between the muscle and transverse process. The same procedure was performed bilaterally.

*Patient 3*. The same procedure was performed in the lateral position. The transverse process and erector spinae muscle were more visible on the upper side of the patient.

## 3. Discussion

ESP was first described having been used for the successful treatment of thoracic neuropathic pain [1]. Later studies demonstrated that ESP was an effective analgesic method in bariatric surgery, pneumothorax surgery, and major abdominal surgery when performed from the thoracic vertebral levels [2–4, 6]. The LA administered during ESP block spreads in the paravertebral space, leading the effective analgesia for somatic and visceral pain [2]. When performed bilaterally ESP block has similar effect as epidural analgesia [2–4].

A cadaver model demonstrated that when 20 ml of fluid was performed at T7 transverse process, the fluid spreads to the level of the C 7-T 2 vertebra levels cranially and L2-3 vertebra levels caudally [7]. ESP block can be performed at T4-5 level for breast and thoracic surgeries and T7-8 levels for abdominal surgeries [3, 7].

Herein we report three cases in which ESP block was successfully performed in patients undergoing various laparoscopic abdominal surgical procedures. Patient 1 and 2 underwent intraoperative ERCP followed by LC. Pain following ERCP is mainly due to visceral pain caused by intestinal distension. Sometimes it can be a painless condition that does not require analgesia, and some surgeons do not even take analgesic order after this procedure. However, there may be differences in pain sensitivity between patients. On the other hand, pain after LC has two causes. The first is visceral pain due to the trauma of gallbladder resection, and the second is parietal pain caused by the skin incision. Other effective analgesic methods have been described for use after LC. Oblique subcostal transversus abdominis plane (OSTAP) block is one of these methods [8, 9]. However, we chose ESP block due to its effect on visceral nerve fibers. We demonstrated that ESP block leads to effective analgesia for both ERCP and LC pain. Frequently oral and intravenous nonsteroidal analgesic drugs (NSAID) are used for LC and are often combined with opioids. Although ESP seems to be a complex regional anesthesia technique for LC and other interventions in combination with LC, regional analgesia techniques can be seen as a technique that can be used to reduce/remove the need for opioid. ESP could been used particularly in patients with comorbidities and/or opioid use, where early mobilization is required. In low pain tolerance patients we could use ESP as a part of multimodal analgesia.

In patient 3, LC and laparoscopic inguinal hernia repair were performed. Preperitoneal CO2 insufflation is also performed for laparoscopic inguinal hernia repair, in contrast to LC. Considering the insufflation time and that laparoscopic surgery was performed for both an upper and lower abdominal pathology, complementary methods to be added to intravenous postoperative analgesia were limited for this patient. TAP block is an option in laparoscopic inguinal hernia repair [10]. We chose to perform ESP block in our patient undergoing both LC and laparoscopic inguinal hernia repair as it has an effect on somatic fibers carrying pain from the surgical field, and visceral fibers carrying pain caused by widespread peritoneal irritation. Bilateral ESP block was observed to be an effective analgesia method.

The combination of paracetamol, NSAID, and opioid in the form of multimodal regimes may be used in the management of postoperative analgesia for both LC and laparoscopic inguinal hernias. However, it should be considered that different regional anesthesia techniques are used for both surgeries and that multislice analgesia plans that are supposed to be supported by regional anesthesia can use only ESP block and provide effective analgesia instead of using more than one regional anesthesia technique. However a heterogeneous case report series is not really a proper exploration of this subgroup, and this is our limitation.

To our knowledge, ESP block has not been reported previously for LC, ERCP, or laparoscopic inguinal hernia repair. ESP block is effective, easy to perform, and can be performed in a short time. Therefore, we believe that bilateral ESP block may have comparable or improved analgesic effect in upper and lower abdominal surgical procedures when compared to other suitable plane blocks. However, further comparative controlled studies are required.

In conclusion, this case series has demonstrated that ESP block can be successfully used in lower and upper abdominal surgical procedures, especially if these procedures are performed in the same session. However, in homogeneous groups, prospective, randomised studies are needed in different surgical procedures to determine the indications, effective practice points, and segments of the ESP block.
